# Supplementary material for: Spatially and temporally defined lysosomal leakage facilitates mitotic chromosome segregation
Source: Nat Commun. 2020 Jan 13;11:229. doi: 10.1038/s41467-019-14009-0 (PMC6957743; doi:10.1038/s41467-019-14009-0)
Supplement: Supplementary file 2 — Description of Additional Supplementary Information [file 41467_2019_14009_MOESM2_ESM.docx]

**Description of Additional Supplementary Files**

**File Name**: Supplementary Movie 1
**Description:** The fate of leaky lysosomes in cytokinesis.

A representative AlexaFluor®488-Dextran-loaded U2OS-mCherry-LGALS3 proceeding from

metaphase to anaphase recorded by live confocal microscopy once per minute under

5 physiological conditions. See Supplementary Fig. 1f for color-adjusted still images. Green,

AlexaFluor®488-Dextran-loaded lysosomes; red, mCherry-LGALS3.

**File Name:** Supplementary Movie 2
**Description:** Chromosome segregation in DMSO-treated control cells.

U2OS-mCherry-α-tubulin/H2B-EGFP cells were treated with DMSO for 2 h upon release from

10 late G2 arrest and recorded by live confocal microscopy every 2 minutes under physiological

conditions. Movies were compiled from merged maximum projections of confocal images with 3

frames/second. Red, mCherry-α-tubulin; green, H2B-EGFP.

**File Name:** Supplementary Movie 3
**Description:** Chromosome segregation errors induced by ConA.

15 U2OS-mCherry-α-tubulin/H2B-EGFP cells were treated with 10 nM ConA for 2 h upon release

from late G2 arrest and recorded by live confocal microscopy every 2 minutes under

physiological conditions. Movies were compiled from merged maximum projections of confocal

images with 3 frames/second. Red, mCherry-α-tubulin; green, H2B-EGFP.

**File Name:** Supplementary Movie 4 **Description:** Chromosome segregation errors induced by Ca-074-Me.

U2OS-mCherry-α-tubulin/H2B-EGFP cells were treated Ca-074-Me for 2 h upon release from

late G2 arrest and recorded by live confocal microscopy every 2 minutes under physiological

conditions. Movies were compiled from merged maximum projections of confocal images with 3

frames/second. Red, mCherry-α-tubulin; green, H2B-EGFP.

**File Name: Supplementary Movie 5
Description:** Chromosome segregation errors induced by CTSB depletion.

U2OS-H2B-GFP cells transfected with control siRNA (a) or CTSB siRNA#2 (b and c) for 72 h

were recorded by live spinning disc confocal microscopy every 2 minutes under physiological

conditions. Movies were compiled from merged maximum projections of confocal images with 6

30 frames/second.
