# Supplementary material for: Spatially and temporally defined lysosomal leakage facilitates mitotic chromosome segregation
Source: Nat Commun. 2020 Jan 13;11:229. doi: 10.1038/s41467-019-14009-0 (PMC6957743; doi:10.1038/s41467-019-14009-0)
Supplement: Supplementary file 3 — Supplementary data [file 41467_2019_14009_MOESM3_ESM.pdf]

Supplementary information

Spatially and temporally defined lysosomal leakage facilitates mitotic chromosome segregation

by Hämälistö et al.

Supplementary Table 1. Primary and secondary antibodies used.

ANTIBODY	SOURCE, IDENTIFIER	DILUTION
Aurora B (rabbit)	Abcam, ab2254	1:200 (ICC)
Aurora B, P-Tyr-232 (rabbit)	Rockland Immunochemicals 600-401-677	1:100 (ICC)
α-tubulin (rabbit)	Abcam, ab15246	1:5000 (WB); 1:300 (ICC)
α-tubulin, HRP-conjugated (mouse)	Abcam, ab40742 (DM1A)	
ß-actin (mouse)	Abcam, ab20272 (8226)	1:3000 (WB)
Bax (rabbit)	Cell Signaling, Cat# 2772	1:200 (ICC)
Cathepsin B (CTSB) (mouse)	Sigma Aldrich, C6243, clone CB59-4B	1:1000 (WB); 1:200 (ICC)
Cathepsin B (CTSB) (rabbit)	Enzo Life Sciences, CB 59-4B11	1:1000 (WB); 1:200 (PLA)
Centromere (ACA/CREST) (human)	Antibodies Incorporated	
	15-234-0001	1:1000 (ICC)
Cystatin B (rabbit)	Proteintech, 10823-1-AP	1:1000 (WB); 1:200 (ICC)
dsDNA (mouse)	Abcam, ab27156 (35I9)	1:500 (PLA)
GFP (mouse)	Clontech, 632381 (JL-8)	1:1000 (WB)
Histone H2B (rabbit)	Abcam, ab1790	1:1000 (WB)
Histone H3, C-terminus (rabbit)	Sigma Aldrich, PLA0148	1:1000 (WB)
Histone H3, N-terminus (rabbit)	Abcam, ab18521	1:1000 (WB)
Histone H3, P-Ser-10 (rabbit)	Cell Signaling, 3377 (D2C8)	1:5000 (WB); 1:3000 (ICC, IHC, PLA)
Hsc70 (mouse)	Provided by Boris Margulis*	
	Clone N22	1:500 (WB)
IgG human, Alexa Fluor®568-conj. (goat)	Thermo Fisher Scientific, A21090	1:1000 (ICC, IHC)
IgG mouse, Alexa Fluor®488-conj. (goat)	Thermo Fisher Scientific, A11029	1:1000 (ICC, IHC)
IgG mouse, Alexa Fluor®488-conj. (donkey)	Thermo Fisher Scientific, A21202	1:1000 (ICC, IHC)
IgG mouse, Alexa Fluor®568-conj. (goat)	Thermo Fisher Scientific, A11004	1:1000 (ICC, IHC)
IgG mouse, Alexa Fluor®594-conj. (goat)	Thermo Fisher Scientific, A11032	1:1000 (ICC, IHC)
IgG mouse, HRP-conjugated (rabbit)	DAKO, P0260	1:10000 (WB)
IgG rabbit, Alexa Fluor®488-conj. (goat)	Thermo Fisher Scientific, A11008	1:1000 (ICC, IHC)
IgG rabbit, Alexa Fluor®488-conj. (donkey)	Thermo Fisher Scientific, A21206	1:1000 (ICC, IHC)
IgG rabbit, Alexa Fluor®568-conj. (goat)	Thermo Fisher Scientific, A11011	1:1000 (ICC, IHC)
IgG rabbit, HRP-conjugated (goat)	Vector Laboratories, PI-1000	1:10000 (WB)
IgG rat, HRP-conj. (goat)	Thermo Fisher Scientific, 31470	1:10000 (WB)
IgG rat, Alexa Fluor®488-conj. (goat)	Thermo Fisher Scientific, A11006	1:1000 (ICC, IHC)
IgG rat, Alexa Fluor®488-conj. (donkey)	Thermo Fisher Scientific, A21208	1:1000 (ICC, IHC)
IgG rat, Alexa Fluor®568-conj. (goat)	Thermo Fisher Scientific, A11077	1:1000 (ICC, IHC)
Lamin B (rabbit), HRP-conjugated	Abcam, ab194109	1:300 (WB)
LAMP1 (rabbit)	Abcam, ab24170	1:200 (ICC, IHC)
LAMP1 (mouse)	Dev. Studies Hybridoma Bank	1:200 (ICC, IHC)
	Clone H4A3	1:200 (IHC)
LAMP2 (mouse)	Dev. Studies Hybridoma Bank	
	Clone H4B4	1:200 (ICC)
LGALS3 (rat)	Provided by Hakon Leffer**	
	Anti-Mac	1:100 (ICC in U2OS, IHC); 1:200 (ICC in MCF7)

TERF1 (mouse)	Abcam, ab10579 (TRF-78)	1:100 (ICC)
TERF2 (rabbit)	Sigma Aldrich, PLA0144	1:2000 (WB)

*Russian Academy of Sciences, St. Petersburg, Russia
**Lund University, Lund, Sweden

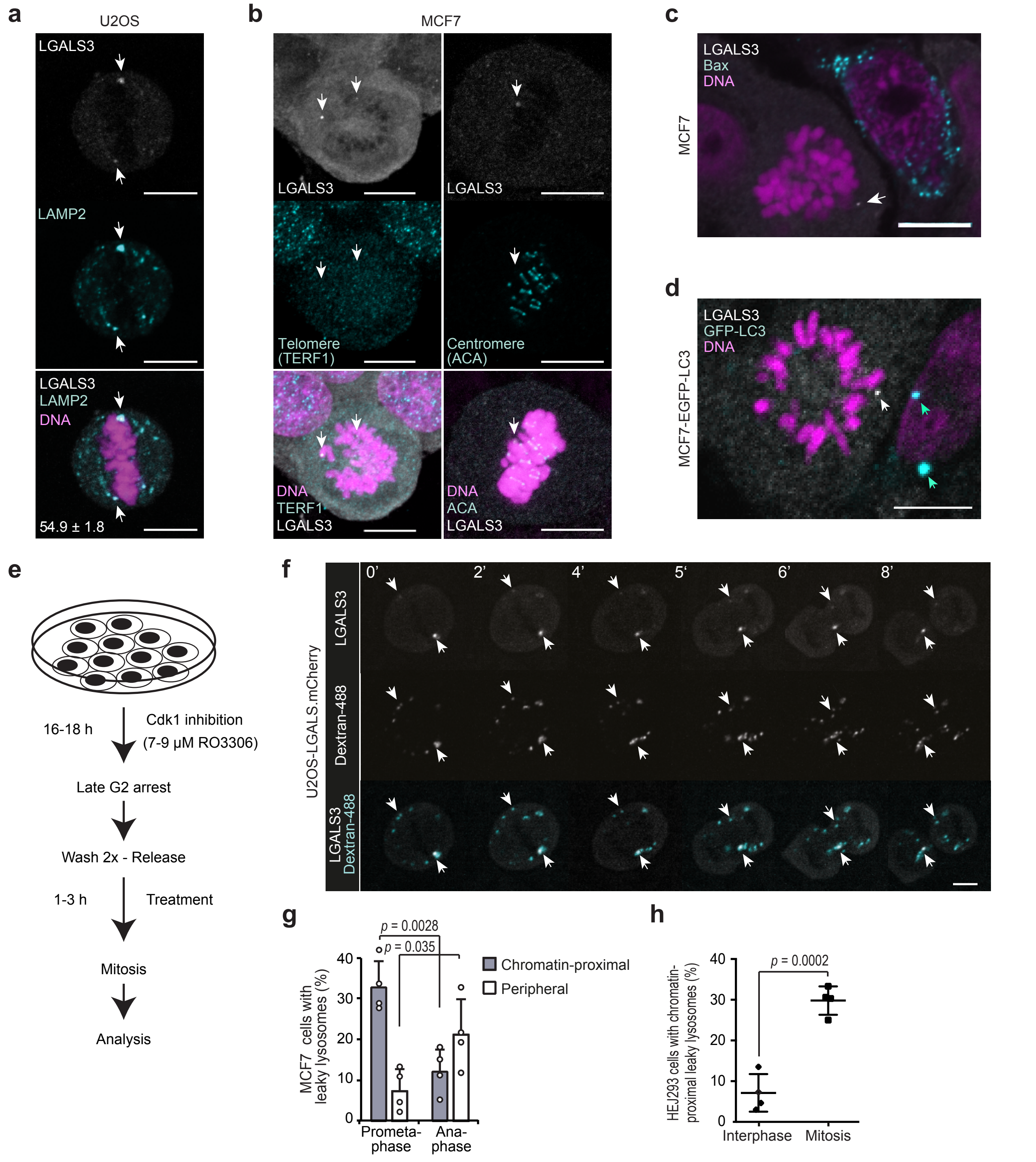

Supplementary Figure 1. Leakage of telomere-proximal lysosomes during mitosis *in vitro*.
(**a**) Representative confocal images of U2OS cells in metaphase stained for LGALS3, LAMP2 and DNA (Hoechst 33342). Value, mean percentage of metaphases with chromatin-proximal leaky lysosomes ±SD, n = 3 independent experiments with 129 metaphases analyzed.
(**b**) Representative confocal images of MCF7 cells in metaphase stained for LGALS, telomeres (TERF1) or centromeres (ACA) and DNA, n = 3 independent experiments with >100 cells analyzed.
(**c**) Representative confocal images of a mitotic (*left*) and an apoptotic (*right*) MCF7 cell stained for LGALS3, Bax and DNA. No Bax puncta were observed in LGALS3 puncta positive metaphase cells studied, n = 3 independent experiments with >100 cells analyzed. The percentage of apoptotic cells was < 1%.
(**d**) Representative confocal images of MCF7-EGFP-LC3 cells in prometaphase (*left*) or interphase (*right*) stained for LGALS3 and DNA. The majority of chromatin proximal leaky lysosomes in pro(metaphase) cells did not co-localize with EGFP-LC3-postive autophagic membranes, n = 3 independent experiments with >100 cells analyzed.
(**e**) Synchronization protocol.
(**f**) Still images of Supplementary Movie 1 showing a representative AlexaFluor®488-Dextran-loaded U2OS-mCherry-LGALS3 cell proceeding from metaphase to cytokinesis, n = 2 independent movies with >10 mitoses observed.
(**g**) Quantification of chromatin-proximal and peripheral leaky lysosomes in MCF7 cells in indicted phases of mitosis. Bars, mean +SD, n = 4 independent experiments with ≥49 prometaphases and ≥42 anaphases analyzed in each experiment.
(**h**) Quantification of chromatin-proximal leaky lysosomes in synchronized HEK293 cells stained for LGALS3, LAMP1 and DNA. Dot blot, mean ±SD, n = 4 independent experiments with >70 interphases and >80 mitoses analyzed in each experiment.
White arrows, chromatin-proximal, leaky lysosomes. Cyan arrows, autophagic membranes.
Scale bars, 10 µm. *P* values by unpaired, two-tailed Students t-test (**g**). Source data for panels **a**, **g** and **h** are provided as Source Data files.

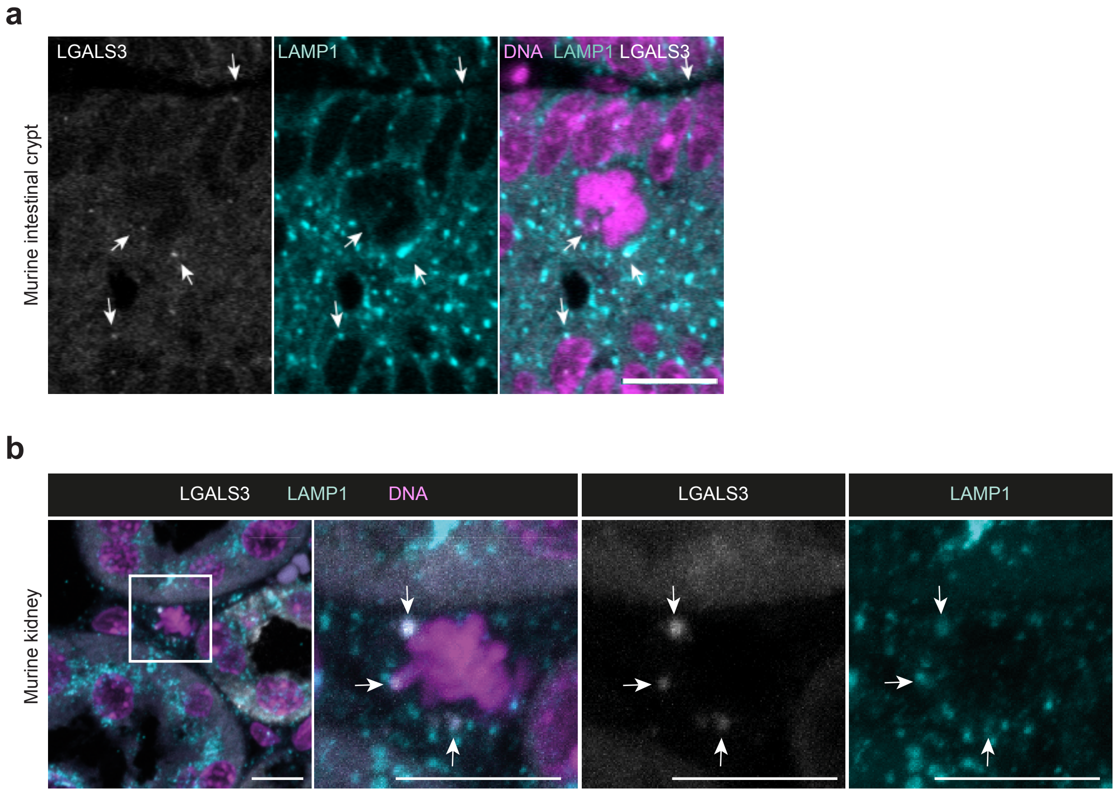

Supplementary Figure 2. Leakage of chromatin-proximal lysosomes during mitosis *in vivo.*
(**a**) Representative confocal images of murine intestinal crypts stained for LGALS3, LAMP1 and DNA, n = 3 mice with 3 non-consecutive sections analyzed for each mouse. Note the co-localization of LGALS puncta and LAMP1.
(**b**) Representative confocal projection images of murine (C57Bl/6) kidney section (4 μm) stained as in (**a**), n = 3 mice with two sections analyzed for each mouse. White square, an interstitial kidney cell magnified in three close-ups. Arrows, chromatin-proximal leaky lysosomes.
Scale bars, 10 μm.

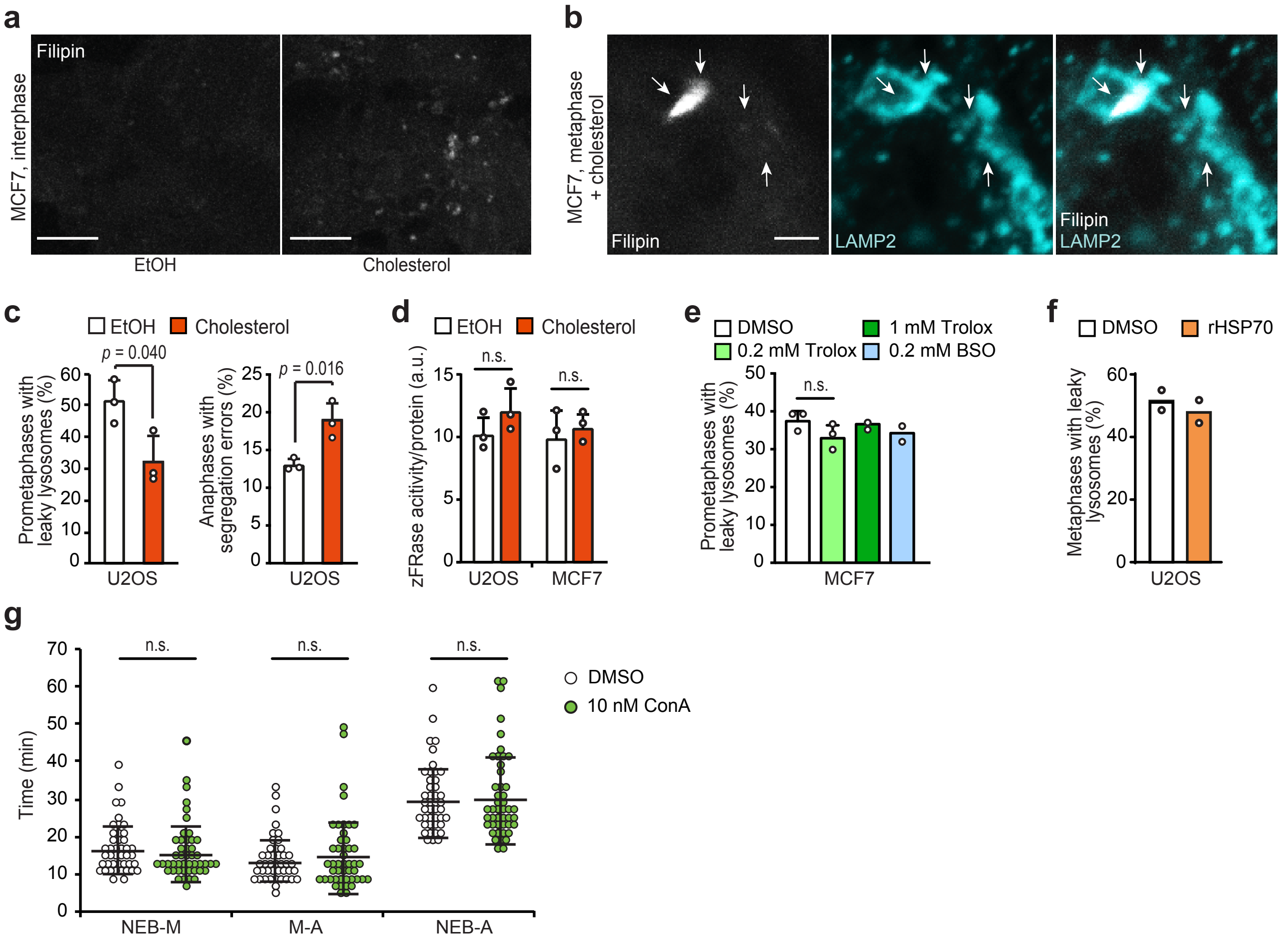

Supplementary Figure 3. Inhibition of lysosomal leakage during mitotic entry causes chromosome segregation errors.
(**a**) Representative confocal images of MCF7 cells treated with vehicle (EtOH) or 30 µM cholesterol for 2 h and stained for cholesterol with Filipin III, n = 2 independenet experiments.
(**b**) Representative confocal images of MCF7 cells treated as in (**a**) upon release from late G2 arrest and stained for cholesterol (filipin III) and LAMP2, n = 3 independenet experiments. Arrows, cholesterol-loaded lysosomes.
(**c**) Quantification of metaphases with chromatin-proximal leaky lysosomes and anaphases with chromatin segregation errors in U2OS cells treated with vehicle (EtOH) or 30 µM cholesterol for 90 min upon release from late G2 arrest. Bars, mean +SD, n = 3 independent experiments with ≥32 prometaphases and ≥56 anaphases analyzed in each experiment.
(**d**) Cysteine cathepsin activity (zFRase) in lysates of U2OS and MCF7 cells treated with vehicle (EtOH) or 30 µM cholesterol for 90 min upon release from late G2 arrest. Bars, mean +SD, n = 3 independent triplicate experiments.
(**e**) Quantification of prometaphases with chromatin-proximal leaky lysosomes in MCF7 cells treated with vehicle (DMSO), indicated concentrations of trolox or buthionine sulfoximine (BSO) for 24 h. Bars (DMSO and 0.2 mM Trolox), mean +SD, n = 3 independent experiments with ≥69 prometaphases analyzed for each sample. Bars (1 mM Trolox and BSO), mean, n = 2 independent experiments with >70 cells analyzed for each condition.
(**f**) Quantification of metaphases with chromatin-proximal leaky lysosomes in U2OS cells treated with vehicle (DMSO) or rHSP70 for 72 h. Bars, mean, n = 2 indpendent triplicate experiments with >20 cells analyzed for each sample.
(**g**) Quantification of the time from nuclear envelope breakdown to metaphase (NEB-M), from metaphase to the onset of anaphase (N-A) and from from nuclear envelope breakdown to the onset of anaphase (NEB-A) in U2OS-GFP-CENP-A/ mCherry-α-tubulin cells treated with DMSO or 10 nM ConA 1-2 h prior to time-lapse imaging. Dot plots, mean ±SD of 43 (DMSO) or 46 (ConA) cells analyzed in 8 independent experiments.
Scale bars, 10 µm.
P values were calculated by unpaired, two-tailed Students t-test (**c**, **d**, **e**, **g**).
Source data for panels **c-g** are provided as Source Data files.

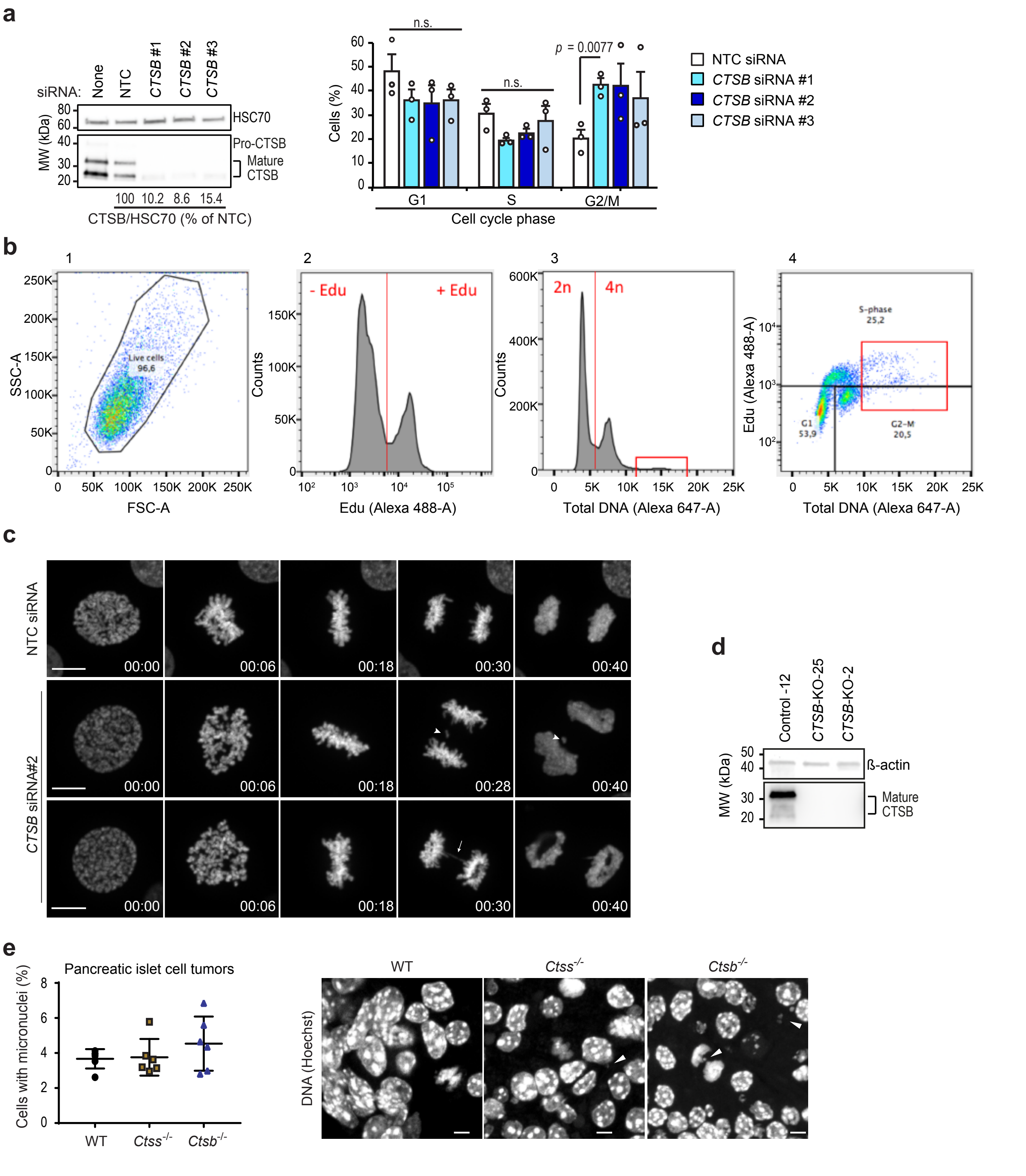

Supplementary Figure 4. Depletion of CTSB causes mitotic defects and nuclear abnormalities.
(**a**) Representative immunoblots of CTSB and HSC70 (left, n = 3 independent experiments) and cell cycle distribution analyzed by 2-dimesional flow cytometry (right) of U2OS cells transfected with control siRNA (NTC) or indicated CTSB siRNAs for 72 h. The values show the CTSB/HSC70 intensity ratios as percentages of the ratio in NTC-transfected cells (left). Bars, mean +SEM, n = 3 independent experiments with >10000 cells analyzed *per* sample (right).
(**b**) Gating used for two-dimesional flow cytometry in (**a**).
(**c**) Representative spinning-disk confocal live-cell images of Movies 5A-C showing U2OS-H2B-GFP cells transfected with the indicated siRNAs for 72 h, n = 8 independent experiments. Arrowheads, lagging chromosomes; arrows, anaphase bridges. Time, h:min.
(**d**) Representative immunoblots of CTSB and ß-actin (loading control) in indicated U2OS CRISPR clones, n > 3 independent experiments.
(**e**) Quantification micronuclei in pancreatic islet cell tumors from RIP1-Tag2 mice crossed with indicated mouse strains (left) and representative images of tumors. Dot blots, mean ±SD, n = 6 mice with >1500 cells analyzed for each tumor sample. Arrow heads, micronuclei.
P values were calculated by unpaired, two-tailed Students t-test. Source data for **a**, **d** and **e** provided as Source Data files.

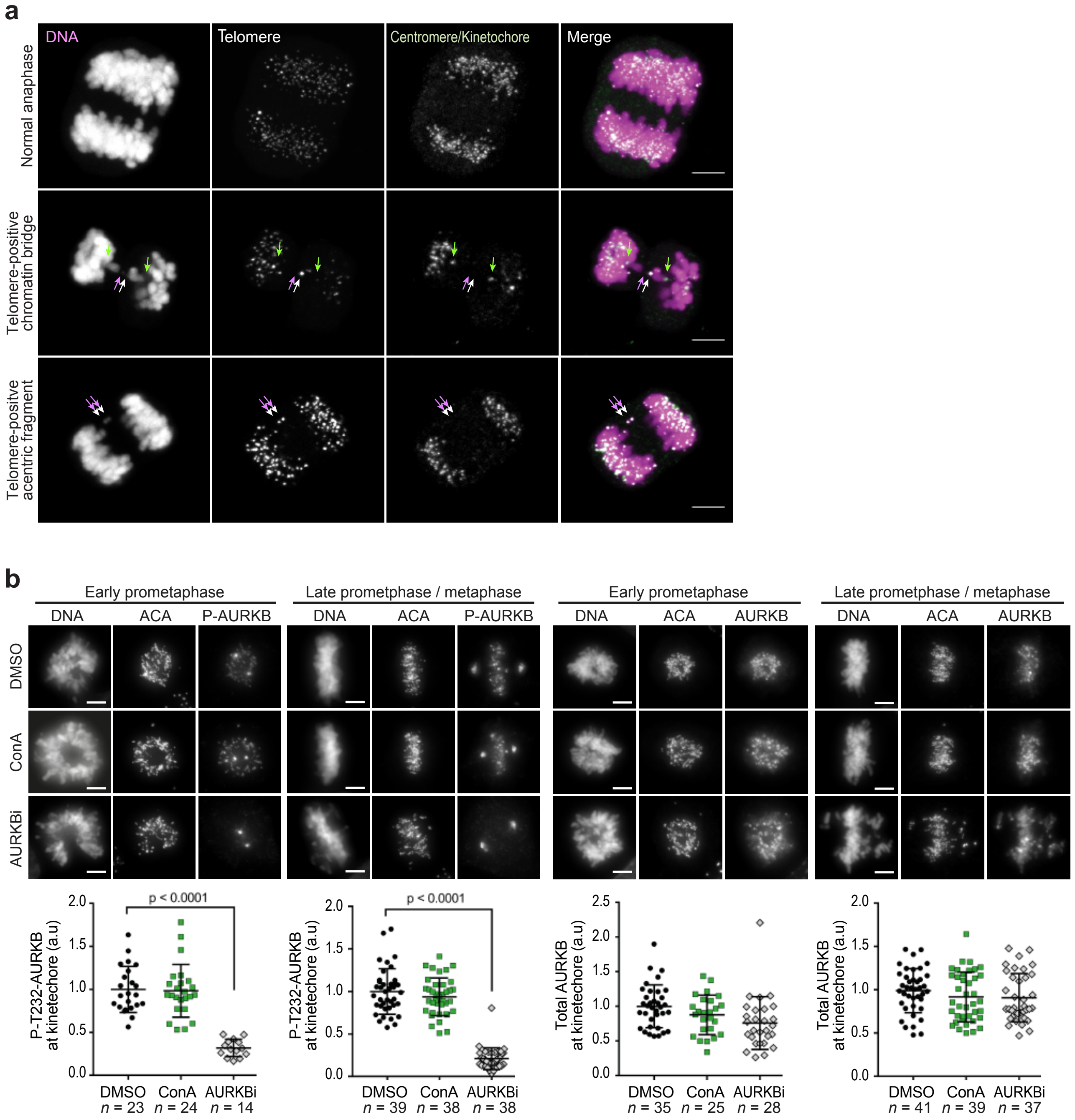

Supplementary Figure 5. Characterization of mitotic defects caused by lysosomal inhibitors.

(**a**) Representative confocal images of a normal anaphase (top), anaphases with a telomere-positive chromatin bridge (middle) or an acentric fragment (bottom) in U2OS cells treated with DMSO (top) or 10 nM ConA (middle and bottom) 1 h upon release from late G2 arrest and stained for kinetochores (ACA), telomeres (immunoFISH) and DNA (Hoechst), n = 3 independent experiments.

(**b**) Representative confocal images and quantification of P-T232-AURKB (P-AURKB) and total AURKB levels at early prometaphase and late prometaphase / metaphase kinetochores of U2OS cells treated with DMSO, 10 nM ConA or 4 µM AURKB inhibitor (AURBKi; ZM-447439) for 2 h. The quantification is based on the fluorescence intensity of anti-P-T232-AURKB or anti-AURKB staining overlapping with anti-centromere staining (ACA). DNA was counterstained with DAPI. Dot plots, mean ±SD, n ≥14 cells analyzed with fluorescence intensity for each condition in 2 independent experiments. *P* values were calculated by unpaired, two-tailed Students t-test. Source data are provided as a Source Data file.

Scale bars, 5 µm.

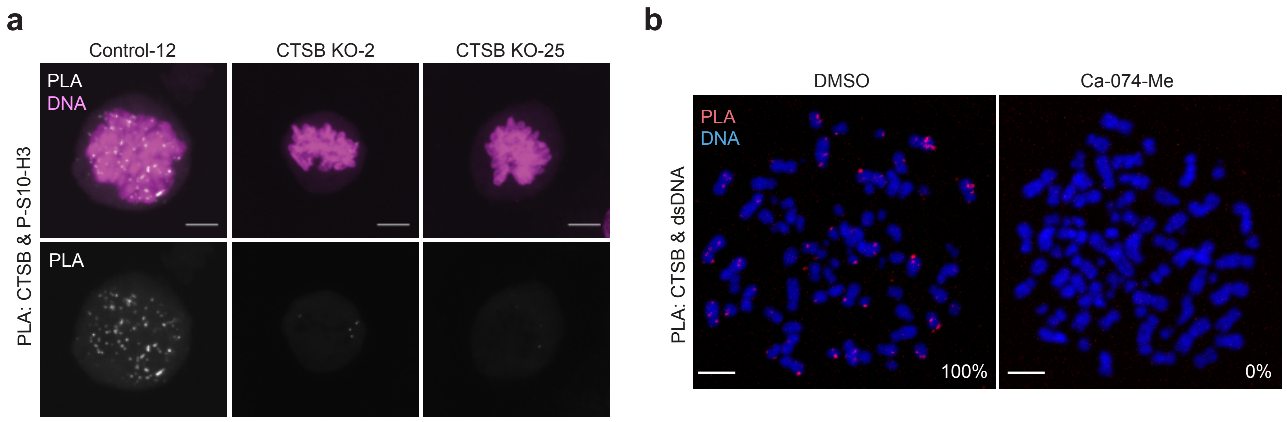

Supplementary Figure 6. CTSB decorates metaphase chromatin.
(**a**) Representative images of PLA puncta with antibodies against CTSB and P-S10-H3 in indicted U2OS CRISPR clones in prometaphase, n = 3 independent experiments.
(**b**) Representative images of PLA puncta with antibodies against CTSB and dsDNA in chromosome spreads from U2OS cells treated with DMSO or 1 μM Ca-074-Me for 1 h upon release from late G2 arrest. Values, percentages of PLA positive cells, n = 2 independent experiments with >8 spreads analyzed.
Scale bars, 5 μm.

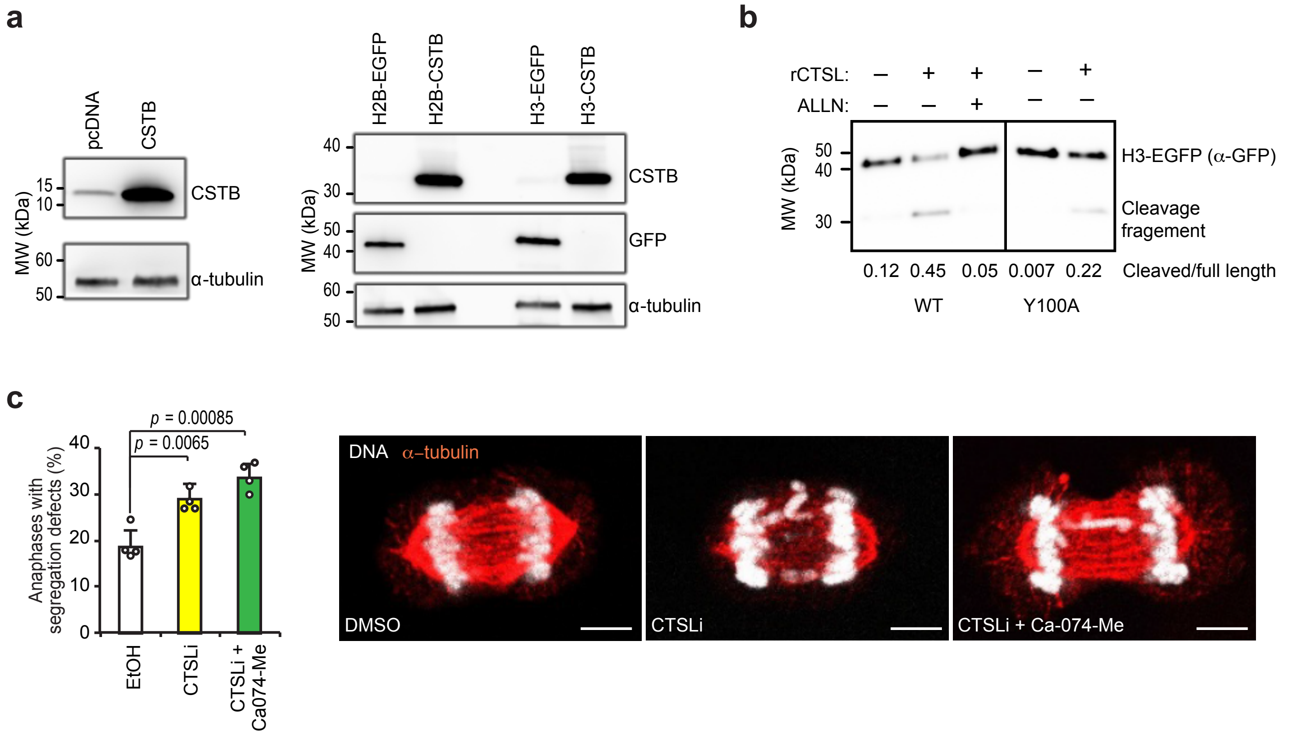

Supplementary Figure 7. Cathepsin-mediated cleavage of histone H3 promotes chromosome segregation.
(**a**) Representative immunoblots of indicated proteins in lysates of U2OS cells transfected as in Fig. 7a, n = 3 independent experiments.
(**b**) Representative immunoblot of H3-EGFP and its cleavage product in chromatin extracts of U2OS cells transfected with pBOS-H3-EGFP with wild type (WT) or Y100A mutant histone H3 for 48 h, n = 3 independent experiments. Chromatin extracts were incubated as indicated with vehicle, rCTSL and 200 nM ALLN for 15 min at 37°C, at pH 7.0.
(**c**) Quantification (left) and representative images of chromosome segregation defects in U2OS cells in anaphase after treatment with DMSO or 0.5 μM CTSL inhibitor IV (CTSLi) alone or with 1 μM Ca-074-Me upon release from late G2 arrest. Bars, mean +SD, n = 4 independent experiments with >80 cells analyzed for each condition. *P* values were calculated by unpaired, two-tailed Students t-test.
Source data for panels **a-c** are provided as Source Data files.
