# Supplementary material for: Spatially and temporally defined lysosomal leakage facilitates mitotic chromosome segregation
Source: Nat Commun. 2020 Jan 13;11:229. doi: 10.1038/s41467-019-14009-0 (PMC6957743; doi:10.1038/s41467-019-14009-0)
Supplement: Supplementary file 9 — Reporting Summary [file 41467_2019_14009_MOESM9_ESM.pdf]

nature research

Corresponding author(s): Marja Jäättelä
Last updated by author(s): Dec 1, 2019

Reporting Summary

Nature Research wishes to improve the reproducibility of the work that we publish. This form provides structure for consistency and transparency in reporting. For further information on Nature Research policies, see Authors & Referees and the Editorial Policy Checklist .

Statistics

For all statistical analyses, confirm that the following items are present in the figure legend, table legend, main text, or Methods section.

n/a	Confirmed	
☐	☒	The exact sample size (*n*) for each experimental group/condition, given as a discrete number and unit of measurement
☐	☒	A statement on whether measurements were taken from distinct samples or whether the same sample was measured repeatedly
☐	☒	The statistical test(s) used AND whether they are one- or two-sided<br>*Only common tests should be described solely by name; describe more complex techniques in the Methods section.*
☒	☐	A description of all covariates tested
☐	☒	A description of any assumptions or corrections, such as tests of normality and adjustment for multiple comparisons
☐	☒	A full description of the statistical parameters including central tendency (e.g. means) or other basic estimates (e.g. regression coefficient) AND variation (e.g. standard deviation) or associated estimates of uncertainty (e.g. confidence intervals)
☐	☒	For null hypothesis testing, the test statistic (e.g. *F*, *t*, *r*) with confidence intervals, effect sizes, degrees of freedom and *P* value noted<br>*Give P values as exact values whenever suitable.*
☒	☐	For Bayesian analysis, information on the choice of priors and Markov chain Monte Carlo settings
☒	☐	For hierarchical and complex designs, identification of the appropriate level for tests and full reporting of outcomes
☒	☐	Estimates of effect sizes (e.g. Cohen's *d*, Pearson's *r*), indicating how they were calculated

Our web collection on statistics for biologists contains articles on many of the points above.

Software and code

Policy information about availability of computer code

Data collection	NA
Data analysis	Image J, Zen from 2010 or black edition from 2012, Metamorph, FlowJo, GraphPad Prism version 7.0a, Excel

For manuscripts utilizing custom algorithms or software that are central to the research but not yet described in published literature, software must be made available to editors/reviewers. We strongly encourage code deposition in a community repository (e.g. GitHub). See the Nature Research guidelines for submitting code & software for further information.

Data

Policy information about availability of data

All manuscripts must include a data availability statement. This statement should provide the following information, where applicable:

- Accession codes, unique identifiers, or web links for publicly available datasets
- A list of figures that have associated raw data
- A description of any restrictions on data availability

All relevant data generated or analysed during this study are included in this published article (and its supplementary information files). The source data underlying Figs 1a, 1d, 1e, 2a, 2b, 3a-c, 4a-c, 5b-d, 6a-c, 7b-g, and Supplementary Figs 1a, 1g, 1h, 3c-g, 4a, 4d, 4e, 5b, and 7a-c are provided as a Source Data file.

Field-specific reporting

Please select the one below that is the best fit for your research. If you are not sure, read the appropriate sections before making your selection.

☒ Life sciences ☐ Behavioural & social sciences ☐ Ecological, evolutionary & environmental sciences

Life sciences study design

All studies must disclose on these points even when the disclosure is negative.

Sample size	Cellular image analyses were applied to 10-250 randomly chosen cells / experiment. All experiments were repeated a minimum of 3 times except for Supplementary Fig. 4a, which was repeated twice.
Data exclusions	Experiments where either negative or positive control did not work were excluded. This practice implies to all experiments done in the laboratory. It should also be noted that we had a 2-3 month period when the ConA and CA-074-Me barely induced any segregation errors (Figure 2 b, segregation errors). When the drugs started working again, the effect was significant, but clearly smaller than the original one - thus we have included 6 experiments into the data set to show the variation. We have no explanation for this.
Replication	All experiments were performed a minimum of 3 times except for Suppl. Fig. 4a, which so far has been performed only twice.
Randomization	Samples were allocated into different groups according to genotypes, treatments or experiment conditions.
Blinding	Data collection and analysis were done with same standards and settings among different groups which equals blinding. Furthermore, key experiments were performed independently by a minimum of two scientists.

Reporting for specific materials, systems and methods

We require information from authors about some types of materials, experimental systems and methods used in many studies. Here, indicate whether each material, system or method listed is relevant to your study. If you are not sure if a list item applies to your research, read the appropriate section before selecting a response.

Materials & experimental systems

n/a	Involved in the study	
☐	☒	Antibodies
☐	☒	Eukaryotic cell lines
☒	☐	Palaeontology
☐	☒	Animals and other organisms
☒	☐	Human research participants
☒	☐	Clinical data

Methods

n/a	Involved in the study	
☒	☐	ChIP-seq
☐	☒	Flow cytometry
☒	☐	MRI-based neuroimaging

Antibodies

Antibodies used	All antibodies' information including supplier name and catalog number are recorded in the methods section.
Validation	Validation of all antibodies were done according to manufactures' websites and datasheet. Antibodies against CTSB, TERF1 and TERF2 were additionally validated by appropriate siRNAs.

Eukaryotic cell lines

Policy information about cell lines

Cell line source(s)	The sources of all cell lines are indicated in the methods section.
Authentication	The cell lines were authenciated by ATCC or RNASeq.
Mycoplasma contamination	All cell lines were found negative for mycoplasma using Venor®GeM Classic PCR kit from Minerva Biolabs.
Commonly misidentified lines (See ICLAC register)	All cell lines were found negative for mycoplasma using Venor®GeM Classic PCR kit from Minerva Biolabs.

Animals and other organisms

Policy information about studies involving animals; ARRIVE guidelines recommended for reporting animal research

Laboratory animals	The details of murine tissues used is provided in materials and methods.
Wild animals	The study did not include wild animals.

Field-collected samples

The study did not include samples collected from the field.

Ethics oversight

All animal studies were approved by the local authorities and carried out in accordance with the NIH guidelines and the Protection of Animals Act.

Note that full information on the approval of the study protocol must also be provided in the manuscript.

Flow Cytometry

Plots

Confirm that:

- [x] The axis labels state the marker and fluorochrome used (e.g. CD4-FITC).
- [x] The axis scales are clearly visible. Include numbers along axes only for bottom left plot of group (a 'group' is an analysis of identical markers).
- [x] All plots are contour plots with outliers or pseudocolor plots.
- [x] A numerical value for number of cells or percentage (with statistics) is provided.

Methodology

Sample preparation

U2OS osteosarcoma cells transfected with indicated siRNAs were cells were incubated with 1 uM EdU for 1 hour to label cells in S phase and stained with total DNA stain (Thermo Fisher Scientific, Cat No F10348) before analyses.

Instrument

BD FACSVerse

Software

FlowJo V10

Cell population abundance

Cell debris or dead cells or cell aggregates were excluded by size. Cells analyzed post-sort were more than 90% of total.

Gating strategy

A typical horseshoe-shaped area was gated (Supplementary Fig. 4b).

- [x] Tick this box to confirm that a figure exemplifying the gating strategy is provided in the Supplementary Information.
